# Nickel-Catalyzed Three-Component Unsymmetrical Bis-Allylation of Alkynes with Alkenes: A Density Functional Theory Study

**DOI:** 10.3390/molecules29071475

**Published:** 2024-03-26

**Authors:** Tao Yu, Jingxuan Zhang, Guo Liu, Liangfei Duan, Kun V. Tian, Gregory A. Chass, Weihua Mu

**Affiliations:** 1 Faculty of Chemistry and Chemical Engineering, Yunnan Normal University, Kunming 650092, China; 2 Department of Chemistry and Pharmaceutical Technology, Sapienza University of Rome, Piazzale Aldo Moro 5, 00185 Rome, Italy; kun.tian@uniroma1.it; 3 Mesoscale Engineering Halcyon S.R.L., 00154 Rome, Italy; g.chass@qmul.ac.uk; 4 School of Physical and Chemical Sciences, Queen Mary University of London, London E1 4NS, UK

**Keywords:** nickel catalysis, NHC, three-component reaction, mechanism, selectivities, dual descriptors, density functional theory (DFT)

## Abstract

Density functional theory (DFT) characterizations were employed to resolve the structural and energetic aspects and product selectivities along the mechanistic reaction paths of the nickel-catalyzed three-component unsymmetrical bis-allylation of alkynes with alkenes. Our putative mechanism initiated with the in situ generation of the active catalytic species [Ni(0)L_2_] (L = NHC) from its precursors [Ni(COD)_2_, NHC·HCl] to activate the alkyne and alkene substrates to form the final skipped trienes. This proceeds via the following five sequential steps: oxidative addition (OA), *β*-F elimination, ring-opening complexation, C-B cleavage and reductive elimination (RE). Both the OA and RE steps (with respective free energy barriers of 24.2 and 24.8 kcal·mol^−1^) contribute to the observed reaction rates, with the former being the selectivity-controlling step of the entire chemical transformation. Electrophilic/nucleophilic properties of selected substrates were accurately predicted through dual descriptors (based on Hirshfeld charges), with the chemo- and regio-selectivities being reasonably predicted and explained. Further distortion/interaction and interaction region indicator (IRI) analyses for key stationary points along reaction profiles indicate that the participation of the third component olefin (allylboronate) and *^t^*BuOK additive played a crucial role in facilitating the reaction and regenerating the active catalyst, ensuring smooth formation of the skipped triene product under a favorably low dosage of the Ni(COD)_2_ catalyst (5 mol%).

## 1. Introduction

Multi-component reactions (MCRs) are an important strategy for the concise and efficient synthesis of complex molecules [1,2], including transition metal-catalyzed MCRs that remain established as a ‘hot topic’ [3,4,5,6]. In particular, nickel-catalyzed MCRs, due to the low cost, availability and special properties of nickel [7,8], continue to generate attention [9,10,11,12]. Yet, Ni-based MCRs still face many challenges such as (1) overcoming competitive and side reactions among multiple components and (2) effective control of chemo-, regio- and stereo-selectivities of the reaction [13,14]. Detailed computational characterizations provide atomistic details from which to raise awareness and understanding of MCRs towards helping overcome these challenges [15,16,17]. Yet, even for seasoned computational scientists, accurate determinations of structure and energetics along the reaction profiles of the putative mechanisms, towards predicting selectivities of MCRs, require significant computational resources, replete with technical difficulties and non-facile interpretation of the result trends. Hence, the provision of accurate reaction-selectivity predictions through relatively low-cost computations is of great significance to both computational and experimental developments in this and related areas [18,19].

**Scheme 1:**
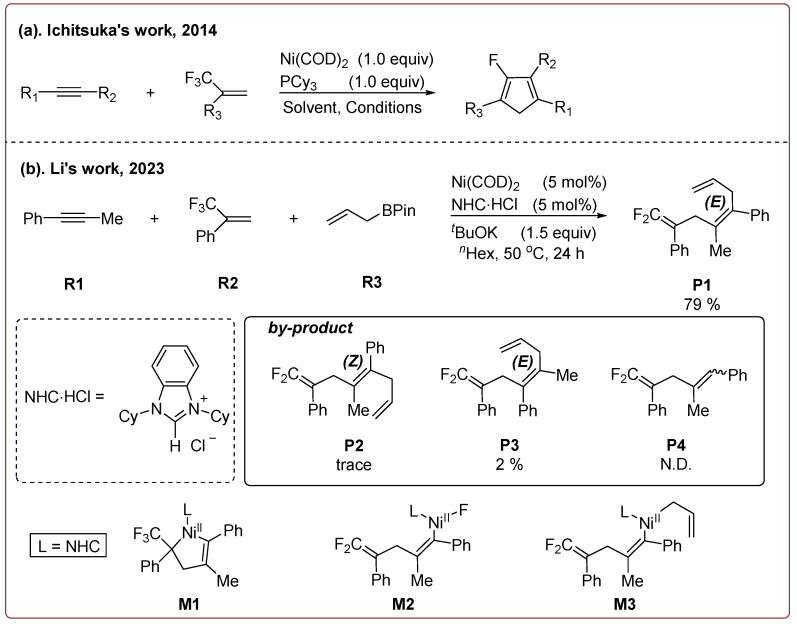
Overview of selected Ni-catalyzed two-component (**a**) [20] and three-component reactions (**b**) [14].

In 2014, Ichitsuka and co-workers prepared 2-fluoro-1,3-cyclopentadienes under mild conditions by using trifluoromethyl olefins and alkynes as substrates, assisted by Ni(0)/PCy_3_ species (Scheme 1a) [20]. This reaction can lead to cyclopentadiene frameworks directly and regioselectively, yet it requires 1.0 equiv of the nickel catalyst. Recently, Li and co-workers synthesized skip trienes (**P1**) by employing trifluoromethyl alkenes (**R1**), alkynes (**R2**) and allylboronate (**R3**) as substrates, realizing good yields (up to 83%) and high regio- and stereo-selectivities under mild conditions with the help of a Ni(0)/NHC catalytic system (Scheme 1b) [14]. It involved a third component (allylboronic acid pinacol ester) and potassium *t*-butoxide (*^t^*BuOK) additive, featuring a low dosage (5 mol%) of the nickel catalyst Ni(COD)_2_; it was favorable relative to Ichitsuka’s 1.0 equiv.

Li et al. speculated that the reaction may proceed by the following putative mechanism: substrates **R1** and **R2** undergo cyclization to form intermediate **M1** under the help of catalysts (Scheme 1), then **M1** experiences *β*-F elimination and is converted into intermediate **M2**, which coordinates with substrate **R3**, assisted by a base additive. Subsequently, intermediate **M3** is generated through the removal of a -BPin group, after which the final product **P1** is produced through reductive elimination of **M3**. However, this could be considered as being overly speculative due to the omission of the essential pre-catalysis process, while also failing to justify the high selectivities exhibited in the reaction. The participation of the third component **R3** and *^t^*BuOK, as well as the significant changes in products and in the amount of catalysts, indicate that the mechanism of this reaction, especially the catalyst recycling mechanism, differs significantly with respect to the two-component reaction. However, the roles of the third component (allylboronic acid pinacol ester) and *^t^*BuOK additive, as well as the mechanistic bases for the pronounced reduction in catalyst dosage (from 1.0 equiv in Ichitsuka’s two-component reaction to 5 mol% in Li’s three-component reaction), are currently unclear, which hinders and limits the rapid development of MCRs.

Towards resolving the relevant atomistic aspects of MCRs (structure, energetics and specificity) and tackling the issues outlined above, we conducted computational studies on the reaction shown in Scheme 1b by employing the density functional theory (DFT) method, with the reaction mechanism (including pre-catalysis and recycling and regeneration of the catalyst) as well as selectivities explored and discussed in detail. Based on confirmation of the rate-determining step (RDS) and selectivity-controlling step (SCS), distortion/interaction analyses [21,22] and interaction region indicator (IRI) analyses [23] were employed to rationalize the high selectivities as well as the role of the base additive. In addition, relatively low-cost computational methods (e.g., dual descriptors [24]) were also used to reliably predict the selectivities of the reaction (Scheme 1b). The emerging good agreement of our theoretical predictions and the corresponding experimental trends makes this work meaningful in helping the organometallic and wider catalysis communities comprehend the mechanisms of such Ni-NHC catalyzed multi-component cyclizations at the molecular level. These trends emerging from relatively low-cost computations provide excellent guidance for experimental chemists in screening substrates and improving MCRs.

## 2. Results and Discussion

### 2.1. Reaction Mechanism

**Figure 1:**
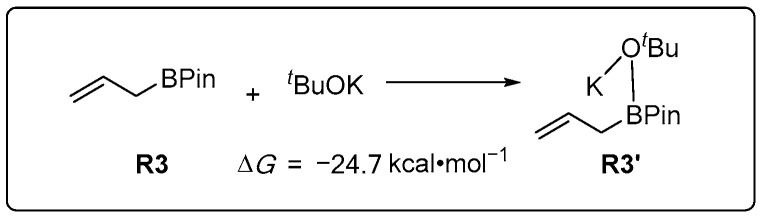
Energetics (Δ*G* in kcal·mol^−1^) for the complexation of **R3** with *^t^*BuOK.

Characterizations of the reaction profile were initiated at the IDSCRF(*^n^*Hex)-PBE0-D3(BJ)/6-311+G(d,p)-SDD(Ni)//IDSCRF(*^n^*Hex)-PBE0-D3(BJ)/6-31+G(d)-SDD(Ni) level and showed that the reaction undergoes the following principal steps: pre-catalysis, oxidative addition (OA), ring-opening complexation and reductive elimination (RE). In addition, the third component **R3** can easily interact with the *^t^*BuOK additive to form a new ‘substrate’ **R3′**, accompanied by a favorable release of 24.7 kcal·mol^−1^ of free energies (Figure 1). This signals that the majority of the unreacted substrate **R3** should exist in the **R3′** form. For reference, **R1** + **R2** + **R3′** is set as the starting point of the catalyzed chemical transformation and assigned a relative free energy of zero (Δ*G* = 0), with all other energetics expressed relative to this.

#### 2.1.1. Pre-Catalysis

**Figure 2:**
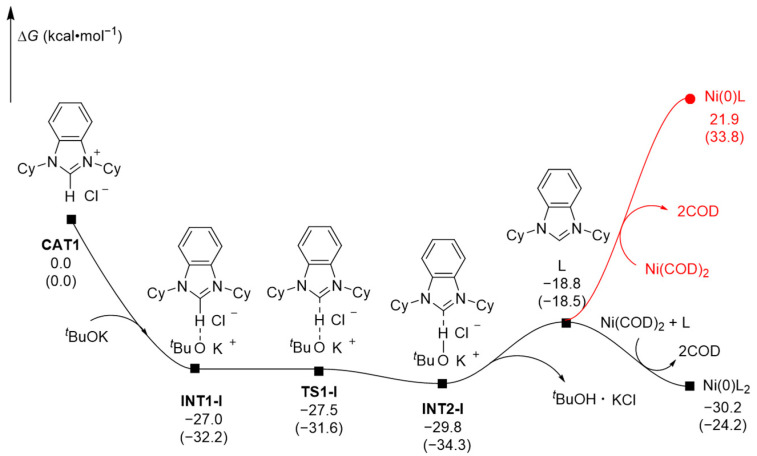
Computed free energy profiles (Δ*G* in kcal·mol^−1^) for the activation of the pre-catalyst, with electronic energies (Δ*E*) noted in parentheses.

The NHC ligand is shown to be cable of capturing protons from alcohols and generating NHC-H^+^ plus alkoxy anions (R-O^−^) [25], which would be the reverse process of the pre-catalysis. To understand the interaction mechanism of the catalyst precursors with the base additive well, we conducted DFT calculations on the pre-catalysis process of the reaction (Figure 2). The catalyst precursor NHC·HCl (**CAT1**) can initially interact with *^t^*BuOK to form the intermediate **INT1-I**, which then undergoes hydrogen migration through transition state **TS1-I**, transforming into intermediate **INT2-I** via a near barrier-free process (ΔΔ*E* = 0.6 kcal·mol^−1^, with a negative ΔΔ*G*). Afterwards, **INT2-I** removes the *^t^*BuOK·KCl fragments and forms the NHC ligand (L), which then undergoes ligand exchange with Ni(COD)_2_ to exothermically generate the active catalytic species Ni(0)L_2_ (L = NHC), releasing 30.2 kcal·mol^−1^ of free energies. On the contrary, the formation of Ni(0)L is endothermic and requires an absorption of 21.9 kcal·mol^−1^ of free energies, indicating that the formation of Ni(0)L is almost uncompetitive to the formation of Ni(0)L_2_. Namely, Ni(0)L_2_ is more stable than Ni(0)L, confirming the active catalytic species in this reaction is Ni(0)L_2_ rather than Ni(0)L.

#### 2.1.2. Oxidative Addition (OA)

**Figure 3:**
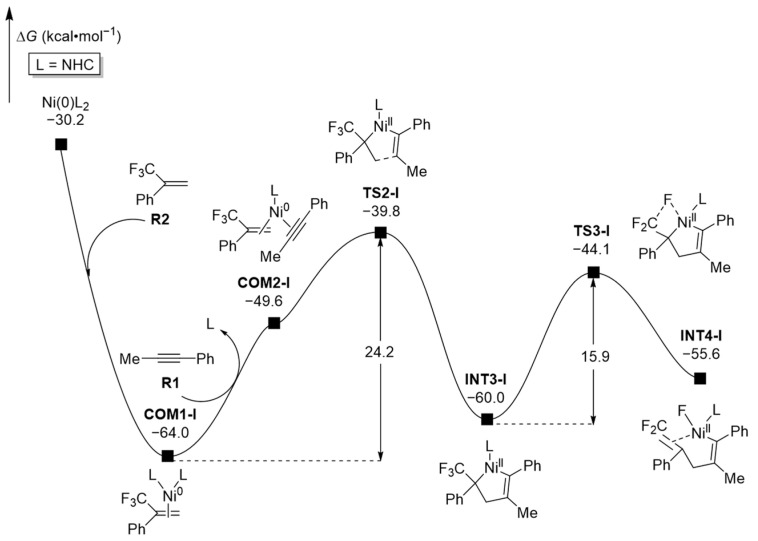
Computed free energy profiles (Δ*G* in kcal·mol^−1^) for the oxidative addition of **R1** with **R2**.

After the generation of the active catalytic species Ni(0)L_2_, the oxidative addition (OA) between substrates **R1** and **R2** was evaluated at the IDSCRF(*^n^*Hex)-PBE0-D3(BJ)/6-311+G(d,p)-SDD(Ni)//IDSCRF(*^n^*Hex)-PBE0-D3(BJ)/6-31+G(d)-SDD(Ni) level (Figure 3). The active species Ni(0)L_2_ quickly coordinates with substrate **R2** to form complex **COM1-I**, releasing 33.8 kcal·mol^−1^ of free energies. **COM1-I** then reacts with substrate **R1** and removes one molecule of ligand **L**, transforming into complex **COM2-I** (Figure S1 in Supplementary Materials for other possible formation pathways of **COM2-I**), which subsequently undergoes oxidative addition (OA) through transition state **TS2-I**, surmounting a total free energy barrier of 24.2 kcal·mol^−1^ (**COM1-I** → **COM2-I** → **TS2-I**) to form a five-membered ring intermediate **INT3-I** (**M1** in Scheme 1), which is feasible at the experimental temperature of 50 °C. Afterwards, intermediate **INT3-I** undergoes *β*-F elimination through transition state **TS3-I** and forms the intermediate **INT4-I**, overcoming a relatively low free energy barrier of 15.9 kcal·mol^−1^ (8.3 kcal·mol^−1^ lower than that of **TS2-I**), signaling that the oxidative addition step is the controlling process of the reaction rate at this stage. If the asymmetric alkene (**R2**) and alkyne (**R1**) coordinate with the nickel center in complex **COM1-I** in different orientations, complexes with different regioselectivities will be formed (see Section 2.2).

#### 2.1.3. Ring-Opening Complexation

**Figure 4:**
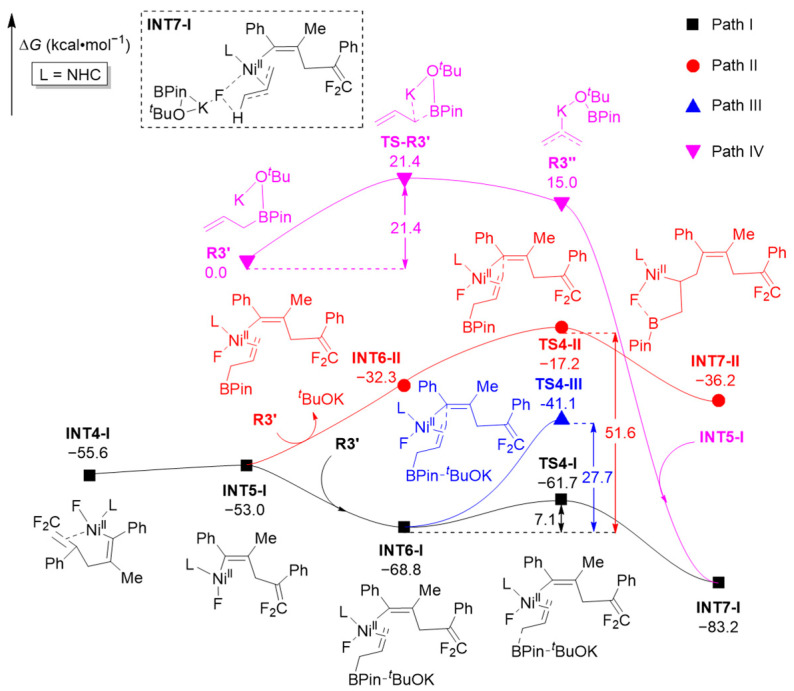
Computed free energy profiles (Δ*G* in kcal·mol^−1^) for the ring-opening complexation process.

As shown in Figure 4, the five-membered ring intermediate **INT4-I** formed through the oxidative addition process can endothermically undergo C-C single bond rotation (ΔΔ*G* = 2.6 kcal·mol^−1^) to convert into ring-opened intermediate **INT5-I** (**M2** in Scheme 1), which is in a more favorable configuration for the subsequent formation of product **P1**. Intermediate **INT5-I** on Path I will then interact with **R3′** (formed through interaction of the third component **R3** with *^t^*BuOK, Figure 1) to form intermediate **INT6-I**, which then undergoes C-B cleavage through transition state **TS4-I** (ΔΔ*G* = 7.1 kcal·mol^−1^) to form the more stable intermediate **INT7-I**. The whole process releases 27.6 kcal·mol^−1^ of free energies and should be easily achievable at an experimental temperature of 50 °C.

Alternatively, if intermediate **INT5-I** reacts with **R3′** and removes *^t^*BuOK (i.e., *^t^*BuOK does not participate in the reaction), the reaction may undergo C-C coupling along Path II, transforming **INT5-I** into intermediate **INT7-II** through transition state **TS4-II**. However, the total free energy barrier of **TS4-II** (**INT6-I** → **INT5-I** → **INT6-II** → **TS4-II**, 51.6 kcal·mol^−1^) is 44.5 kcal·mol^−1^ higher than that of **TS4-I** (7.1 kcal·mol^−1^), which indicates null possibility for the reaction to occur along Path II (see Figure S2 for subsequent details of Path II). Similarly, if intermediate **INT6-I** does not undergo C-B cleavage but experiences C-C coupling and reductive elimination directly through transition state **TS4-III**, the reaction must surmount a free energy barrier of 27.7 kcal·mol^−1^, which is 20.6 kcal·mol^−1^ higher than **TS4-I**. This suggests that Path I is much more preferable than Path III in energy. In addition, the possibility of **R3′** undergoing C-B cleavage through transition state **TS-R3′** to transform into the intermediate **R3″**, and then reacting with intermediate **INT5-I** to transform into intermediate **INT7-I** (Path IV), was also investigated. It was found that the free energy barrier of **TS-R3′** (21.4 kcal·mol^−1^) is 14.3 kcal·mol^−1^ higher than **TS4-I**, and all stationary points on Path IV are significantly less stable than those along Path I. Therefore, the reaction is most likely to proceed along Path I at this stage.

#### 2.1.4. Reductive Elimination (RE)

**Figure 5:**
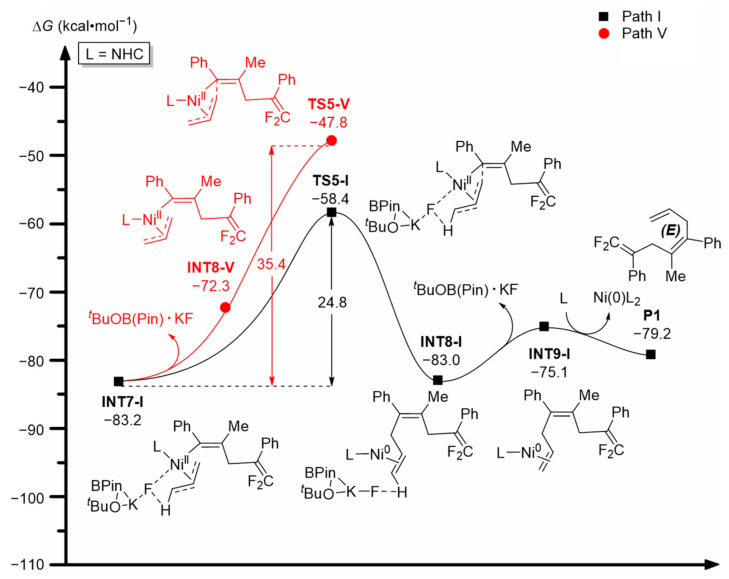
Computed free energy profiles (Δ*G* in kcal·mol^−1^) for the reductive elimination process.

As shown in Figure 5, the intermediate **INT7-I** formed will undergo C-C coupling and reductive elimination through transition state **TS5-I**, overcoming a free energy barrier of 24.8 kcal·mol^−1^ and transforming into intermediate **INT8-I**. Afterwards, **INT8-I** removes the *^t^*BuOB(Pin)·KF fragment and converts to intermediate **INT9-I**. Finally, **INT9-I** interacts with ligand L and forms the product **P1**, accompanied by the regeneration and recycling of the active catalytic species Ni(0)L_2_ (L = NHC). If intermediate **INT7-I** removes the *^t^*BuOB(Pin)·KF fragment and transforms to **INT8-V** (**M3** in Scheme 1) first (Path V), the following C-C coupling and reductive elimination process must overcome a total free energy barrier as high as 35.4 kcal·mol^−1^ (**INT7-I** → **INT8-V** → **TS5-V**), which is ~10.6 kcal·mol^−1^ higher than that for Path I (24.8 kcal·mol^−1^). This indicates that the reaction would barely proceed along Path V at an experimental temperature of 50 °C, and Path I clearly dominates the formation of product **P1**.

**Figure 6:**
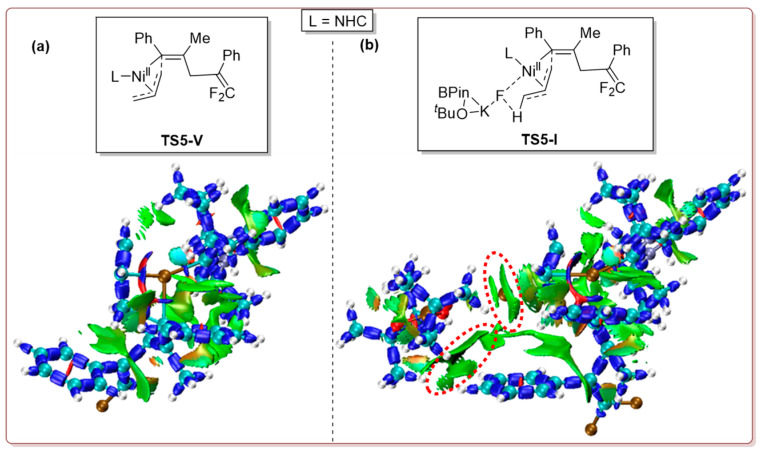
Interaction region indicator (IRI) analyses for **TS5-V** (**a**) and **TS5-I** (**b**).

IRI analyses of transition states **TS5-I** and **TS5-V** show significant van der Waals interaction (Figure 6, in green) between the leaving fragment (*^t^*BuOB(Pin)·KF) and the remaining part of transition state **TS5-I**, translating to **TS5-I** being a more stable structure than **TS5-V**. Additionally, negligible steric hindrance (in red) can be seen in both molecular graphics, which suggests that the electronic effect is the key factor determining the reaction rate here.

**Figure 7:**
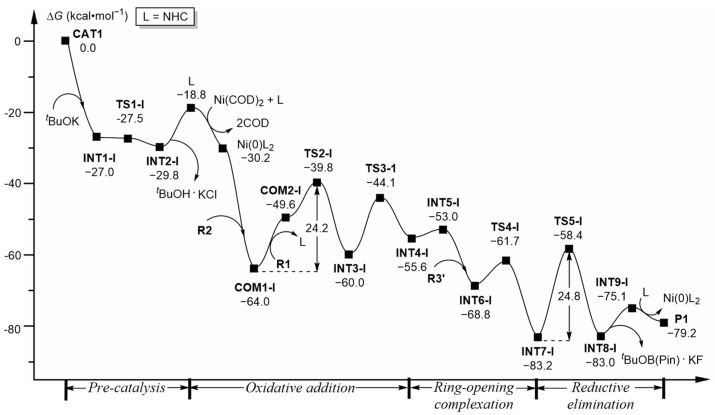
Computed free energy profiles (Δ*G* in kcal·mol^−1^) for the whole reaction process.

In summary, the final product **P1** should be generated as follows: the catalyst precursors (Ni(COD)_2_ and NHC·HCl) initially interact with the *^t^*BuOK additive, which undergoes hydrogen migration and generates the active catalytic species Ni(0)L_2_ (L = NHC) in situ after removal of the *^t^*BuOH·KCl fragment (Figure 7). Then, Ni(0)L_2_ activates alkyne and olefin substrates (**R2** and **R1**), undergoes oxidative addition to transform into the five-membered ring intermediate **INT3-I**, followed by *β*-F elimination and converts into intermediate **INT4-I**. Afterwards, **INT4-I** experiences ring-opening through C-C single bond rotation to complex with **R3′**, leading to intermediate **INT7-I** through C-B cleavage. Finally, **INT7-I** undergoes C-C coupling and reductive elimination, removes the *^t^*BuOB(Pin)·KF fragment and regenerates the active species Ni(0)L_2_, while forming product **P1**. The free energy barriers for the oxidative addition and reductive elimination processes are 24.2 (**TS2-I**) and 24.8 (**TS5-I**) kcal·mol^−1^, respectively, both higher than others and indicating their reaction rate-controlling characteristics. Half-lives transferred from the above two steps’ barriers are 0.66 h and 1.69 h, respectively, while the reaction times delivered from the energy span approximation formula proposed by Kozuch and Shaik [26] are 0.96 h and 2.44 h, respectively, with both being reasonably consistent with the experimental reaction time (~24 h) and illustrating that these kinetic predictions are reliable.

### 2.2. Selectivities

#### 2.2.1. Chemoselectivities

Based on Fu and Cao’s reports, the Hirshfeld charges and dual descriptors Δ*f* can accurately predict the active sites of electrophilic and nucleophilic reagents [18,19]. Hence, the Hirshfeld charges [27] and dual descriptors Δ*f* [24] (based on Hirshfeld charges) of various substrates were measured for evaluating their nucleophilic/electrophilic reaction activities, as well as for exploring the chemoselectivities of this reaction. Regardless, olefin **R3** can interact with the *^t^*BuOK additive easily to form **R3′** (Figure 1) and **R3′** may transform into **R3″** further (Figure 4); **R3′** and **R3″** were also taken into consideration (Table 1). For molecules with multiple reaction sites, the most nucleophilic/electrophilic sites are evaluated for their reaction activities.

As shown in Table 1, the dual descriptors (Δ*f*) of the C1 and C2 sites in substrate **R1** are both negative, with that of the C1 site being lower. Contrarily, the Δ*f* of the C3 and C4 sites in substrate **R2** are both positive, with that of the C4 site being higher. This illustrates that **R1** is a nucleophile while **R2** is an electrophile. Similar to substrate **R1**, the dual descriptors of all sites in **R3**, **R3′** and **R3″** are negative, with that of the C5, C7 and C9 sites being lower in each of them and indicating that **R3**, **R3′** and **R3″** are also nucleophiles. Furthermore, the dual descriptors (Δ*f*) of C5, C7 and C9 in **R3**, **R3′** and **R3″** are smaller than C1 in **R1**, which suggests that **R1** is the weakest nucleophilic reagent (Lewis base). With the exception of **R2**, the nucleophilic/electrophilic reactivity predictions based on Hirshfeld charges are basically consistent with that delivered from the dual descriptors (Table 1). According to the calculated dual descriptors, the reaction between substrate **R2** and **R1** is most favorable, as **R2** is a weak Lewis acid while **R1** is a weak Lewis base. Further natural population analysis (NPA) results show that there are −0.05189 and 0.03468 charges populated on the C1 and C2 atoms in substrate **R1**, respectively (Table S1), while the NPA charges distributed on the C3 and C4 atoms in substrate **R2** are −0.13534 and −0.31486, respectively. Thus, when **R1** reacts with **R2**, the C2 atom in **R1** is more likely to interact with the C4 atom in **R2**, leading to the experimentally detected main product **P1**.

The energetics for different substrates complexing with the nickel catalyst were further evaluated at the IDSCRF(*^n^*Hex)-PBE0-D3(BJ)/6-311+G(d,p)-SDD(Ni)//IDSCRF (*^n^*Hex)-PBE0-D3(BJ)/6-31+G(d)-SDD (Ni) level, with the most stable conformations presented in Table 2 (see Figure S3 for other conformations arising from varying orientations of functional groups). The relative stabilities of different complexes are consistent with the predictions derived from the dual descriptors (Δ*f*) as a whole: substrate **R2** is an electrophilic reagent, while other substrates are nucleophilic reagents. Therefore, the complexes involving substrate **R2** are relatively more stable than others, with the one formed between substrate **R2**, **R1** and the nickel catalyst being most stable (Entry 1 in Table 2). Clearly, the dual descriptors (Δ*f*, Table 1) provide substantially accurate predictions for relative free energies of each complex, although it considers only the electronic structure of the substrates [18] while ignoring steric hindrance effects and other factors such as interactions in the forming complex, evidencing that electronic effects are the principal controlling factor for the reaction selectivities observed.

Trends in the free energy barrier can also validate the above predicted chemoselectivities quite well. As shown in Figure 3, the energy barrier for the oxidative addition of complex **COM2-I** formed by the complexation of substrate **R2** with **R1** and the nickel catalyst is 9.8 kcal·mol^−1^ (**TS2-I**), which is lower than that of the complex formed by the complexation of substrate **R2** with **R3**/**R3′** and the nickel catalyst (19.9/27.7 kcal·mol^−1^, Figure S3), also lower than that of the complex formed by the complexation of substrate **R1** with **R3** and the nickel catalyst (20.6 kcal·mol^−1^). For the complex formed by the complexation of substrate **R1** with **R3′** and the nickel catalyst (Entry 5 in Table 2), multiple attempts failed in locating any corresponding transition states, perhaps due to the fact that both **R1** and **R3′** are nucleophiles and it is electronically difficult for them to react with each other. In short, the complex **COM2-I** formed between substrate **R2**, **R1** and the nickel catalyst is the most prone one in the oxidative addition step; this is consistent with previous predictions based on the dual descriptors (Δ*f*, Table 1) and with the experimentally detected chemoselectivities.

#### 2.2.2. Regioselectivities

**Figure 8:**
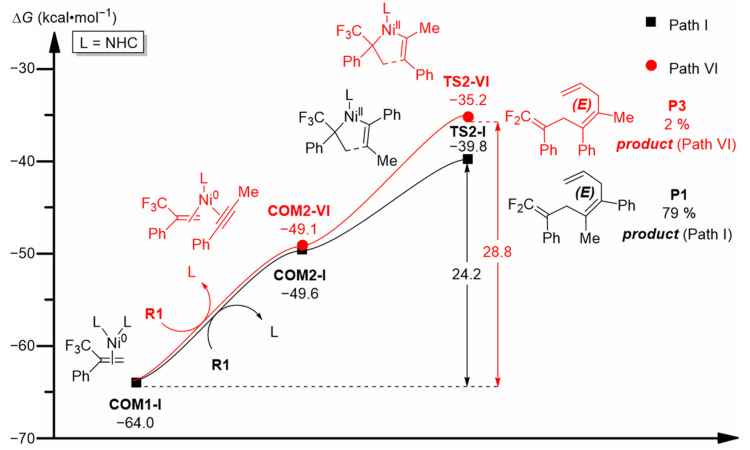
Computed free energy profiles (Δ*G* in kcal·mol^−1^) for Path VI, with that of Path I noted (in black) for comparison.

As mentioned in Section 2.1.2, the oxidative addition step (**COM2-I** → **TS2-I**, Figure 3) has a regioselectivity-determining role in this reaction. When the asymmetric alkyne (**R1**) complexes with the nickel center in complex **COM1-I** in different orientations, another complex **COM2-VI** may be formed (Figure 8), which is of comparable stability to **COM2-I**. Nevertheless, the oxidative addition of **COM2-VI** requires overcoming a total free energy barrier of 28.8 kcal·mol^−1^ (**COM1-I** → **COM2-VI** → **TS2-VI**), which is 4.6 kcal·mol^−1^ higher than that of **TS2-I** on Path I (24.2 kcal·mol^−1^), justifying the dominant formation of product **P1** along Path I. The regioselectivity predictions based on energetic results are in complete agreement with corresponding experimental results (79% of **P1** detected at an experimental temperature of 50 °C, while only 2% of the regioisomer **P3** could be detected), and they are also consistent with the predictions based on dual descriptors (the C1 site in **R1** is more susceptible to the attack of the electrophilic nickel center).

### 2.3. Recycling and Regeneration of Catalyst

**Figure 9:**
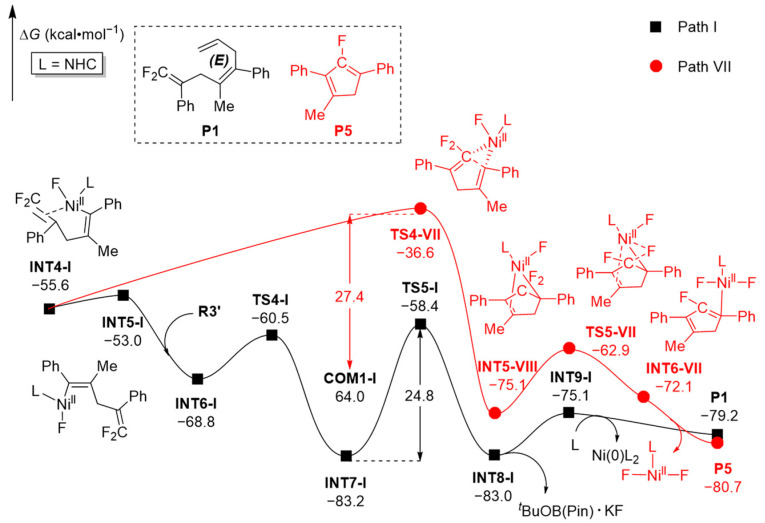
Computed free energy profiles (Δ*G* in kcal·mol^−1^) for Path VII, with that of Path I noted (in black) for comparison.

Experiments [14,20] showed that the participation of the third component **R3** and the *^t^*BuOK additive significantly reduced the catalyst dosage (from 1.0 equiv to 5 mol%) compared to its two-component alternative. Towards resolving the bases of this reduction in catalyst loading, as well as the recycling and regeneration mechanism of the catalyst, we conducted computations on the corresponding two-component reaction (Path VII in Figure 9) at the same level to ensure comparability. In absence of the third component **R3** and the *^t^*BuOK additive, intermediate **INT4-I** will undergo C-C coupling (through **TS4-VII**) along path VII to generate the five-membered ring intermediate **INT5-VII**, by surmounting a free energy barrier of 27.4 kcal·mol^−1^. Subsequently, intermediate **INT5-VII** accomplishes *β*-F elimination through transition state **TS5-VII** and converts into intermediate **INT6-VII**, which may remove a Ni(II)F_2_L fragment to generate the final product **P5**. However, in light of the fact that the energy barrier of **TS4-VII** (27.4 kcal·mol^−1^) is 2.6 kcal·mol^−1^ higher than that of the local maximum **TS5-I** on Path I (24.8 kcal·mol^−1^), this two-component reaction should be more difficult with respect to the three-component one, albeit it can also still proceed under the corresponding experimental conditions [20].

**Figure 10:**
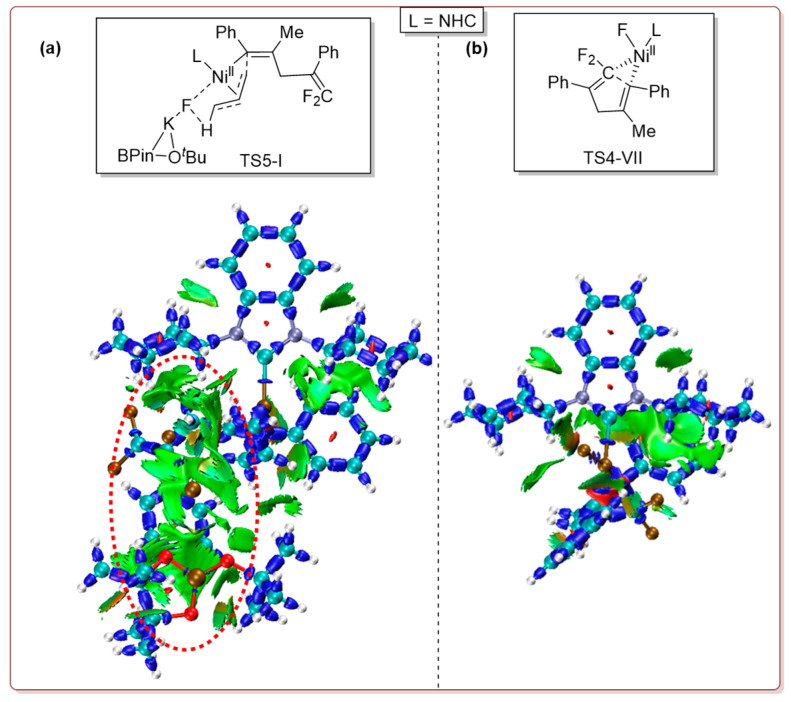
Interaction region indicator (IRI) analyses for **TS5-I** (**a**) and **TS4-VII** (**b**).

Distortion/interaction analyses results on transition states **TS5-I** and **TS4-VII** are shown in Table 3. The distortion energies of each fragment as well as the total distortion energies of **TS5-I** (Δ*E*_dist_) are all higher than that of **TS4-VII**. Meanwhile, the interaction energies (Δ*E*_int_) of **TS5-I** are significantly lower than that of **TS4-VII**. This means that transition state **TS5-I** displays more pronounced distortions yet stronger interactions compared to **TS4-VII**, and the dual effects make it more stable than **TS4-VII**. The IRI analyses results of transition states **TS5-I** and **TS4-VII** (Figure 10) reveal pronounced van der Waals interactions (in red box) between the third component **R3** and the *^t^*BuOK fragment with other parts of **TS5-I**, rationalizing **TS5-I**’s raised stability relative to **TS4-VII**. Both the results of energy decomposition and IRI analyses represent well the corresponding experimental result that the three-component reaction involving the *^t^*BuOK additive is easier than its two-component alternative without a base additive.

It can also be seen in Figure 9 that the final product **P5** in the two-component reaction without the *^t^*BuOK additive (Path VII) could be generated accompanied by the removal of the Ni(II)F_2_L fragment; however, it is near impossible for Ni(II)F_2_L to be converted into the active species Ni(0)L_2_ which can catalyze the reaction in the cycle (Figure S4). As a result, the two-component reaction developed by Ichitsuka requires 1.0 equiv of Ni(COD)_2_ to ensure that the reaction proceeds [20]. In contrast, the final product **P1** in the three-component reaction involving the *^t^*BuOK additive (Path I) is formed accompanied by the removal of the active Ni(0)L_2_ species, and Ni(0)L_2_ can participate in subsequent catalytic cycles directly until the end of the reaction. Hence, the three-component reaction developed by Li only requires a catalytic amount of Ni(COD)_2_ catalyst (5 mol%), generating a 79% yield of **P1** under mild conditions [14]. These results are of significance to raise understanding of the catalytic cycle and recycling mechanism of catalysts in transition metal-catalyzed multi-component reactions.

## 3. Computational Methods

Extensive literature on benchmark calculations for density functional theory (DFT) methods show that the PBE0 method combined with D3(BJ) dispersion correction, and the 6-31+G(d) and 6-311+G(d,p) basis sets, perform well and are sufficient for the correct description of geometries and energies in reaction systems involving transition metals, sufficient to reproduce related experimental trends with reasonable accuracy [28,29,30,31]. Thus, all computations in this work were performed using the Gaussian 16 software package [32] by employing the PBE0 method [33] and D3(BJ) [34] dispersion correction. All geometry optimizations and vibrational frequency analyses were conducted by using the 6-31+G(d) [35,36,37] (for C, H, O, N, F, B, and K) and SDD basis sets [38] (for Ni), with corresponding single-point corrections performed employing the 6-311+G(d,p) [35,39,40,41] (C, H, O, N, F, B, and K) and SDD basis sets (for Ni). All stationary points were confirmed as stable points or first-order saddle points (for TSs) on their respective potential energy hypersurfaces. Intrinsic reaction coordinate (IRC) [42,43] analyses were performed on key transition states to ensure their correct connections to correspondent reactants and products. The effect of the *^n^*Hex solvent was taken into account in all computations by using the IDSCRF atomic radii [44], denoted as IDSCRF(*^n^*Hex). All free energies reported have been corrected to experimental temperature (323.15 K) by using the THERMO program [45], to include the translational entropy contributions in solution (*S_trans_*_(*l*)_); the latter is in contrast to the default use of the gas-phase one (*S_trans_*_(*g*)_). Interaction region indicator (IRI) analyses, Hirshfeld charge and dual descriptor (based on Hirshfeld charge) calculations were performed by using the Multiwfn program [46]. Natural population analyses (NPA) were conducted on selected stationary points using the NBO 5.0 program [47].

## 4. Conclusions

Density functional theory (DFT) at the IDSCRF(*^n^*Hex)-PBE0-D3(BJ)/6-311+G(d,p)- SDD(Ni)//IDSCRF(*^n^*Hex)-PBE0-D3(BJ)/6-31+G(d)-SDD(Ni) level was employed to explore the reaction mechanism and selectivities of the nickel-catalyzed three-component asymmetrical bis-allylation of alkynes with alkenes. The following conclusions can be drawn based on present explorations: (1) The reaction proceeds via pre-catalysis, oxidative addition (OA), *β*-F elimination, ring opening complexation and reduction elimination (RE) in sequence, among which OA and RE are both essential in controlling the reaction rates, with free energy barriers being 24.2 and 24.8 kcal·mol^−1^, respectively. (2) The oxidative addition step (**COM2-I** → **TS2-I**) acts as the selectivity-controlling step, with the chemo- and regioselectivity predictions based on the dual descriptors (Δ*f*, based on Hirshfeld charge) and energy barrier calculations being in good accordance with product selectivities observed in experiments. This evidences the dual descriptor and energy barrier calculations as excellent low-cost tools for predicting the selectivities of MCRs and even more complicated reactions. (3) The active Ni(0)L_2_ (L = NHC) species in the three-component reaction involving the *^t^*BuOK additive can be regenerated accompanied by the formation of the final skipped trienes (**P1**), allowing for a catalytic amount of Ni(COD)_2_ catalyst (5 mol%) to generate a 79% yield of **P1** under mild conditions. Further energy decomposition and IRI analyses reveal that the van der Waals interactions between the third component (**R3**) and *^t^*BuOK fragment with other parts in the key stationary point (**TS5-I**) are essential in stabilizing it and thus facilitate the reaction under mild conditions. A reasonable explanation for the improvement in catalyst dosage (from 1.0 equiv to 5 mol%) further makes this work valuable to raise understanding of the catalytic cycle and recycling mechanism of catalysts in transition metal-catalyzed multi-component reactions.

## Figures and Tables

**Table 1:** Hirshfeld charge and dual descriptor (Δ*f*, based on Hirshfeld charge) of key atoms in different substrates, with the nucleophilic sites in red and the electrophilic sites in green.

Compounds		Reaction Sites	Hirshfeld Charge	Δ*f*
**R1**	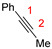	**C1**	−0.07888	−0.05522
		**C2**	−0.05603	−0.04918
**R2**	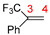	**C3**	−0.02576	0.04212
		**C4**	−0.06044	0.05396
**R3**	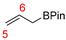	**C5**	−0.10885	−0.18748
		**C6**	−0.02911	−0.13880
**R3′**	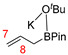	**C7**	−0.12664	−0.09557
		**C8**	−0.02076	−0.01886
**R3″**	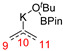	**C9**	−0.25696	−0.20117
		**C10**	−0.08526	−0.03908
		**C11**	−0.24887	−0.19177

**Table 2:** Energetics (in kcal·mol^−1^) for the complexation of different substrates with the nickel catalyst (L = NHC).

Entry	Substrate A	Substrate B	Compounds	Δ*G* (kcal·mol^−1^)
1	**R2**	**R1**	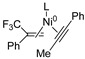	−49.6
2	**R2**	**R3**	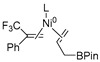	−22.3
3	**R1**	**R3**	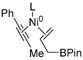	−18.6
4	**R2**	**R3′**	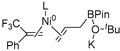	−47.7
5	**R1**	**R3′**	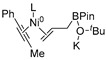	−44.3
6	**R2**	**R3′**	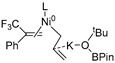	−36.3
7	**R1**	**R3″**	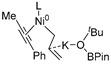	−26.4

**Table 3:** Distortion/interaction analysis results (single-point corrections [*E*(sp)] in kcal·mol^−1^) for **TS5-I** and **TS4-VII** (L = NHC), with Δ*E*_dist_ and Δ*E*_int_ indicating distortion and interaction energies, respectively; ΔΔ*E* = Δ*E*_dist_ + Δ*E*_int_.

TSs	Δ*E*_dist_				Δ*E*_int_	ΔΔ*E*
	Fragment 1	Fragment 2	Fragment 3	Total Δ*E*_dist_		
	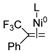	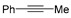	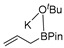			
**TS5-I**	87.5	2.1	40.4	130.0	−138.0	−8.0
**TS4-VII**	35.7	−5.7	0.0	30.0	−10.1	19.9

## Data Availability

The data underlying this study are available in the published article and its Supplementary Materials.

## Supplementary Materials

The following supporting information can be downloaded at https://www.mdpi.com/article/10.3390/molecules29071475/s1, Figure S1: Computed free energy profiles for the generation of **COM2-I**, Figure S2: Computed free energy profiles for Path II, Figure S3: Computed free energies for the complexation of different substrates with the nickel catalyst, Figure S4: Energetics for the transformation of Ni(II)F_2_L to Ni(0)L_2_, Table S1: NPA charges on key atoms in reactants **R1** and **R2**, Table S2: Calculated energy results; Optimized Cartesian coordinates for all stationary points (PDF and XYZ).

## References

[1] Alegre-Requena J., Marqués-López E., Herrera R., Herrera R., Marqués-López E. (2015). IntroductIon: Multicomponent Strategies. Multicomponent Reactions: Concepts and Applications for Design and Synthesis.

[2] Cioc R., Ruijter E., Orru R.V.A. (2014). Multicomponent Reactions: Advanced Tools for Sustainable Organic Synthesis. Green Chem..

[3] Sharma U., Sharma N., Vachhani D., Van der Eycken E.V. (2015). Metal-mediated Post-Ugi Transformations for the Construction of Diverse Heterocyclic Scaffolds. Chem. Soc. Rev..

[4] Zhang J.S., Liu L., Chen T.Q., Han L.B. (2018). Recent Advances in Transition Metal-Catalyzed Three-Component Difunctionalization of Alkenes. Chem. Asian J..

[5] Sharma U., Ranjan P., Van der Eycken E.V., You S.L. (2020). Sequential and Direct Multicomponent Reaction (MCR)-Based Dearomatization Strategies. Chem. Soc. Rev..

[6] John S., Gulati S., Shankaraiah N. (2021). Recent Advances in Multi-Component Reactions and Their Mechanistic Insights: A Triennium Review. Org. Chem. Front..

[7] Tobisu M., Chatani N. (2015). Cross-Couplings Using Aryl Ethers via C−O Bond Activation Enabled by Nickel Catalysts. Acc. Chem. Res..

[8] Bhakta S., Ghosh T. (2021). Nickel-Catalyzed Cascade Reactions. Eur. J. Org. Chem..

[9] Ichitsuka T., Fujita T., Ichikawa J. (2015). Nickel-Catalyzed Allylic C(sp3)−F Bond Activation of Trifluoromethyl Groups via *β*-Fluorine Elimination: Synthesis of Difluoro-1,4-Dienes. ACS Catal..

[10] Pellissier H. (2016). Enantioselective Nickel-Catalyzed Domino and Tandem Processes. Curr. Org. Chem..

[11] Chen J., Zhu S.L. (2021). Nickel-Catalyzed Multicomponent Coupling: Synthesis of α-Chiral Ketones by Reductive Hydrocarbonylation of Alkenes. J. Am. Chem. Soc..

[12] Ding C., Ren Y.Y., Yu Y., Yin G.Y. (2023). Ligand-Modulated Nickel-Catalyzed Regioselective Silylalkylation of Alkenes. Nat. Commun..

[13] Zhang Y.R., Wang H., Mao Y.J., Shi S.L. (2022). Ni-Catalyzed Three-Component Coupling Reaction of Butadiene, Aldimines and Alkenylboronic Acids. Chin. J. Org. Chem..

[14] Li Y., Zhang W.S., Yang S.N., Wang X.Y., Liu Y., Ji D.W., Chen Q.A. (2023). Nickel-Catalyzed Unsymmetrical Bis-Allylation of Alkynes. Angew. Chem. Int. Ed..

[15] Wang X.G., Li Y., Liu H.C., Zhang B.S., Gou X.Y., Wang Q., Ma J.W., Liang Y.M. (2019). Three-Component Ruthenium-Catalyzed Direct Meta-Selective C–H Activation of Arenes: A New Approach to The Alkylarylation of Alkenes. J. Am. Chem. Soc..

[16] Zhang Z., Sabat N., Frison G., Marinetti A., Guinchard X. (2022). Enantioselective Au(I)-Catalyzed Multicomponent Annulation via Tethered Counterion-Directed Catalysis. ACS Catal..

[17] Pannilawithana N., Son M., Hwang D., Baik M.H., Yi C. (2023). Scope and Mechanistic Studies on the Ruthenium-Catalyzed Multicomponent Deaminative C–H Coupling Reaction of Phenols with Aldehydes and Enamines for the Formation of Xanthene and Dioxacyclic Derivatives. ACS Catal..

[18] Fu R., Lu T., Chen F.L. (2014). Comparing Methods for Predicting the Reactive Site of Electrophilic Substitution. Acta Phys. Chim. Sin..

[19] Cao J.J., Ren Q., Chen F.L., Lu T. (2015). Comparative Study on the Methods for Predicting the Reactive Site of Nucleophilic Reaction. Sci. China-Chem..

[20] Ichitsuka T., Fujita T., Arita T., Ichikawa J. (2014). Double C-F Bond Activation through *β*-Fluorine Elimination: Nickel-Mediated [3+2] Cycloaddition of 2-Trifluoromethyl-1-Alkenes with Alkynes. Angew. Chem. Int. Ed..

[21] Bickelhaupt F., Houk K. (2017). Analyzing Reaction Rates with the Distortion/Interaction-Activation Strain Model. Angew. Chem. Int. Ed..

[22] Wolters L., Bickelhaupt F. (2015). The Activation Strain Model and Molecular Orbital Theory. WIREs. Comput. Mol. Sci..

[23] Lu T., Chen F.W. (2021). Interaction Region Indicator: A Simple Real Space Function Clearly Revealing Both Chemical Bonds and Weak Interactions. Chem.-Methods.

[24] Morell C., Grand A., Toro-Labbé A. (2004). New Dual Descriptor for Chemical Reactivity. J. Phys. Chem. A.

[25] Lessa M., Fajardo J., Delarmelina M., Carneiro J. (2021). A DFT Study on the Mechanism for Polymerization of *δ*-Valerolactone Initiated by N-heterocyclic Carbene (NHC) Catalysts. Mol. Catal..

[26] Kozuch S., Shaik S. (2011). How to Conceptualize Catalytic Cycles? The Energetic Span Model. Acc. Chem. Res..

[27] Hirshfeld F.L. (1977). Bonded-Atom Fragments for Describing Molecular Charge Densities. Theor. Chem. Acc..

[28] Michael B., Reimann C., Pantazis D., Bredow T., Neese F. (2008). Geometries of Third-Row Transition-Metal Complexes from Density-Functional Theory. J. Chem. Theory Comput..

[29] Steinmetz M., Grimme S. (2013). Benchmark Study of the Performance of Density Functional Theory for Bond Activations with (Ni,Pd)-Based Transition-Metal Catalysts. ChemistryOpen.

[30] Dohm S., Hansen A., Steinmetz M., Grimme S., Checinski M. (2018). Comprehensive Thermochemical Benchmark Set of Realistic Closed-Shell Metal Organic Reactions. J. Chem. Theory Comput..

[31] Basiuk V., Escobar A., Molina H. (2002). Basis Set Effects on B3LYP Geometries and Energies: Case Study of Interstellar Reaction HN=CH_2_ + •C≡N → H_2_N–C(•)H–C≡N. Int. J. Quantum Chem..

[32] Frisch M.J., Trucks G.W., Schlegel H.B., Scuseria G.E., Robb M.A., Cheeseman J.R., Scalmani G., Barone V., Petersson G.A., Nakatsuji H. (2019). Gaussian 16, Revision C.01.

[33] Adamo C., Barone V. (1999). Toward Reliable Density Functional Methods without Adjustable Parameters: The PBE0 Model. J. Chem. Phys..

[34] Grimme S., Ehrlich S., Goerigk L. (2011). Effect of the Damping Function in Dispersion Corrected Density Functional Theory. J. Comp. Chem..

[35] Clark T., Chandrasekhar J., Spitznagel G.W., Schleyer P. (1983). Efficient Diffuse Function-Augmented Basis Sets for Anion Calculations. III. The 3-21+G Basis Set for First-Row Elements, Li-F. J. Comp. Chem..

[36] Petersson G.A., Bennett A., Tensfeldt T., Al-Laham M.A., Shirley W., Mantzaris J. (1988). A Complete Basis Set Model Chemistry. I. The Total Energies of Closed-Shell Atoms and Hydrides of the First-Row Elements. J. Chem. Phys..

[37] Petersson G.A., Al-Laham M.A. (1991). A Complete Basis Set Model Chemistry. II. Open-Shell Systems and the Total Energies of the First-Eow Atoms. J. Chem. Phys..

[38] Dolg M., Wedig U., Stoll H., Preuss H. (1987). Energyadjusted Ab Initio Pseudopotentials for the First Row Transition Elements. J. Chem. Phys..

[39] McLean A.D., Chandler G.S. (1980). Contracted Gaussian Basis Sets for Molecular Calculations. I. Second Row Atoms, Z = 11–18. J. Chem. Phys..

[40] Krishnan R., Binkley J.S., Seeger R., Pople J.A. (1980). Selfconsistent Molecular Orbital Methods. XX. A Basis Set for Correlated Wave Functions. J. Chem. Phys..

[41] Frisch M.J., Pople J.A. (1984). Selfconsistent Molecular Orbital Methods 25. Supplementary Functions for Gaussian Basis Sets. J. Chem. Phys..

[42] Fukui K. (1970). Formulation of the Reaction Coordinate. J. Phys. Chem..

[43] Fukui K. (1981). The Path of Chemical Reactions—The IRC Approach. Acc. Chem. Res..

[44] Tao J.Y., Mu W.H., Chass G.A., Tang T.H., Fang D.C. (2013). Balancing the Atomic Waistline: Isodensity-Based SCRF Radii for Main-Group Elements and Transition Metals. Int. J. Quantum Chem..

[45] Fang D.-C. (2013). THERMO Program.

[46] Lu T., Chen F.W. (2012). Multiwfn: A Multifunctional Wavefunction Analyzer. J. Comput. Chem..

[47] Glendening E.D., Badenhoop J.K., Reed A.E., Carpenter J.E., Bohmann J.A., Morales C.M., Weinhold F. (2001). NBO 5.0.

