# Supplementary material for: Nickel-Catalyzed Three-Component Unsymmetrical Bis-Allylation of Alkynes with Alkenes: A Density Functional Theory Study
Source: Molecules. 2024 Mar 26;29(7):1475. doi: 10.3390/molecules29071475 (PMC11012778; doi:10.3390/molecules29071475)
Supplement: Supplementary file 1 [file molecules-29-01475-s001.zip › molecules-2923416_SI_mwh-20240326-for pub.pdf]

Supplementary Materials for

Nickel-Catalyzed Three-Component Unsymmetrical Bis-Allylation of Alkynes with Alkenes: A Density Functional Theory Study

Tao Yu[1], Jingxuan Zhang[1], Guo Liu[1], Liangfei Duan[1], Kun V Tian[2,3], Gregory A. Chass[3,4], Weihua Mu[1]'*

[1] Faculty of Chemistry and Chemical Engineering, Yunnan Normal University, Kunming 650092, China;

[2] Department of Chemistry and Pharmaceutical Technology, Sapienza University of Rome, Piazzale Aldo Moro 5, 00185 Rome, Italy

[3] Mesoscale Engineering Halcyon Srl, 00154 Roma RM, Italy

[4] School of Physical and Chemical Sciences, Queen Mary University of London, London E1 4NS, United Kingdom

Table of Contents	Page
**Figure S1**. Computed free energy profiles (Δ*G* in kcal·mol^{-1}) for the generation of **COM2-I**.	S2
**Figure S2**. Computed free energy profiles (Δ*G* in kcal·mol^{-1}) for Path II.	S2
**Figure S3**. Computed free energies (Δ*G* in kcal·mol^{-1}) for the complexation of different substrates with the nickel catalyst, with the the conformations of lowest energies noted in red.	S3
**Figure S4**. Energetics (Δ*G* in kcal·mol^{-1}) for the transformation of Ni(II)F_2L to Ni(0)L_2.	S3
**Table S1**. NPA charges on key atoms in reactants **R1** and **R2**.	S3
**Table S2**. The total energies (*E*: a.u.), zero-point energies (*ZPE*: kcal·mol^{-1}) and Gibbs free energies [*G*(298 K)] for stationary points located at IDSCRF(^{n}Hex)-PBE0-D3(BJ)/6-31+G*-SDD(Ni) level, as well as the single-point corrections [(*E*(sp): a.u.) and Gibbs free energies [*G*(sol,323.15 K): a.u] located at IDSCRF(^{n}Hex)-PBE0-D3(BJ) /6-311+G**-SDD(Ni)//IDSCRF(^{n}Hex)-PBE0-D3(BJ)/6-31+G*-SDD(Ni) level.	S4

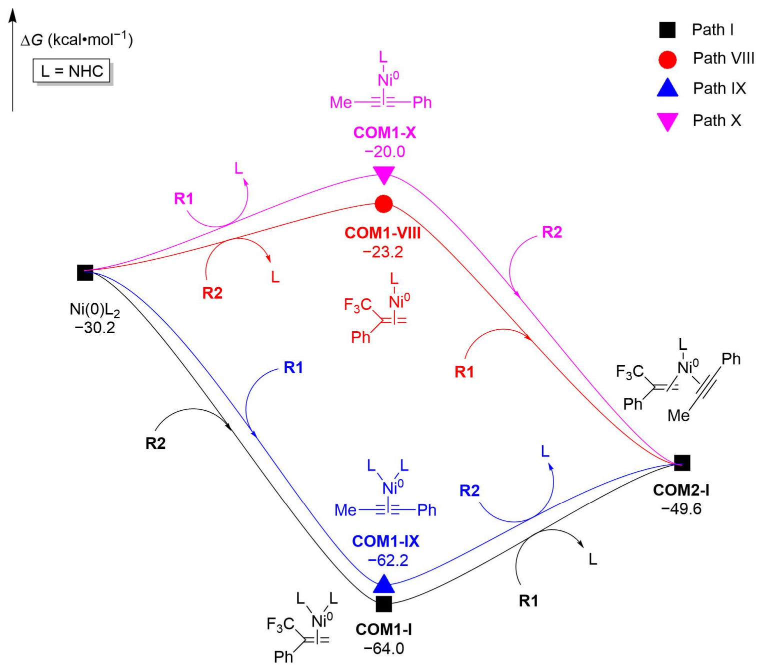

Figure S1. Computed free energy profiles (ΔG in kcal·mol^{-1}) for the generation of **COM2-I**.

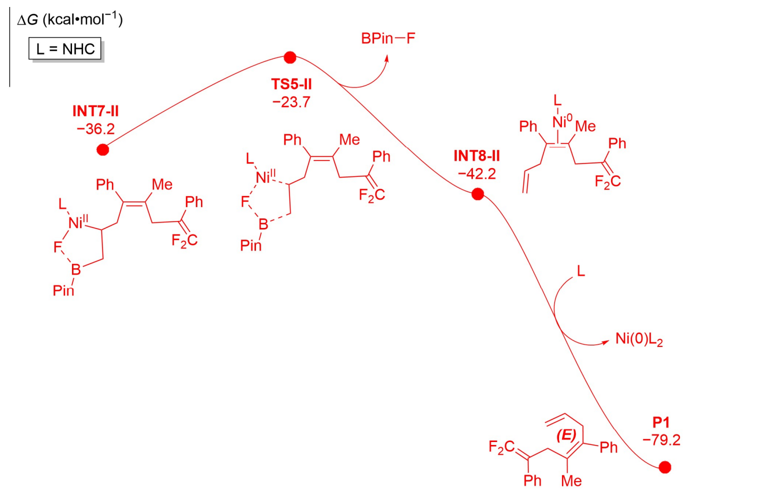

Figure S2. Computed free energy profiles (ΔG in kcal·mol^{-1}) for Path II.

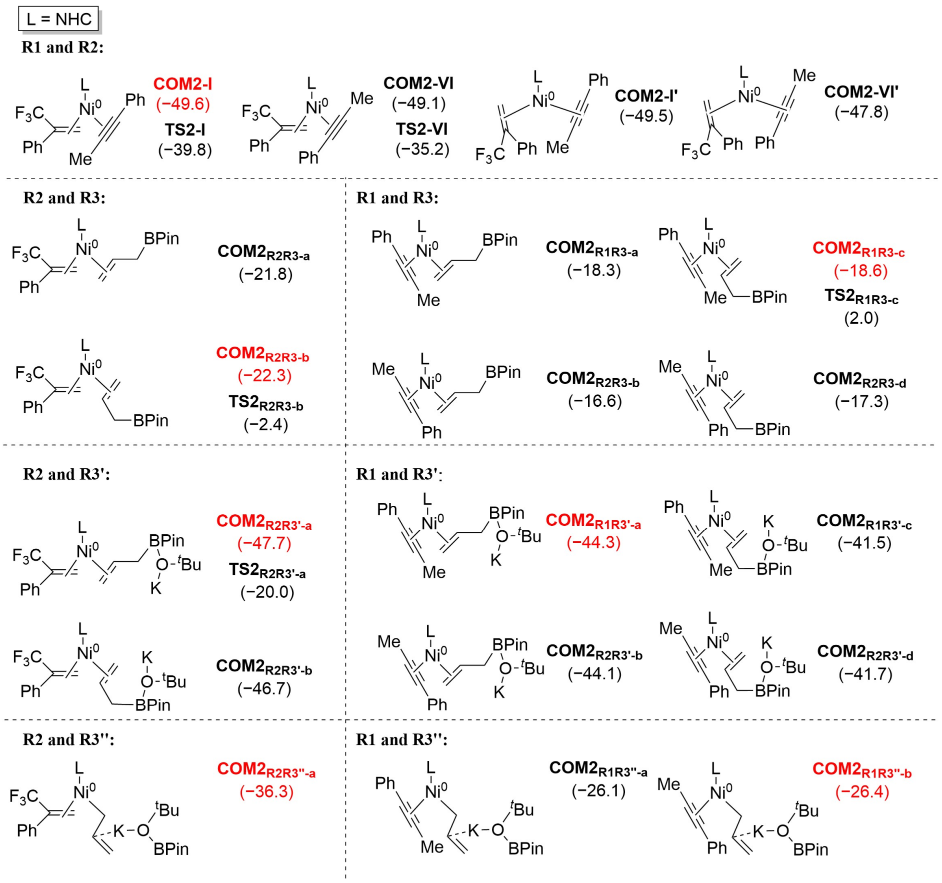

Figure S3. Computed free energies (ΔG in kcal·mol^{-1}) for the complexation of different substrates with the nickel catalyst, with the conformations of lowest energies noted in red.

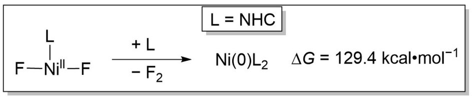

Figure S4. Energetics (ΔG in kcal·mol^{-1}) for the transformation of Ni(II)F_2L to Ni(0)L_2.

Table S1. NPA charges on key atoms in reactants **R1** and **R2**.

Compounds		Reaction sites	NPA charges
**R1**	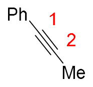	C1	-0.05189
		C2	**0.03468**
**R2**	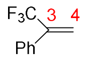	C3	-0.13534
		C4	**-0.31486**

Table S2. The total energies (*E*: a.u.), zero-point energies (*ZPE*: kcal·mol^{-1}) and Gibbs free energies [*G*(298 K)] for stationary points located at IDSCRF(^{n}Hex)-PBE0-D3(BJ)/6-31+G*-SDD(Ni) level, as well as the single-point corrections [(*E*(sp): a.u.) and Gibbs free energies [*G*(sol,323.15 K): a.u] located at IDSCRF(^{n}Hex)-PBE0-D3(BJ)/6-311+G**-SDD(Ni)//IDSCRF(^{n}Hex) -PBE0-D3(BJ)/6-31+G*-SDD(Ni) level.

**Species**	***E***	***ZPE***	***G*(298 K)**	***E*(sp)**	***G*(sol, 323.15 K)**
**CAT1**	-1308.95329	275.05603	-1308.56454	-1309.16806	-1308.77427
^{t}BuOK	-832.56794	77.91958	-832.47743	-832.66657	-832.56797
Ni(COD)2	-794.29783	230.93805	-793.97068	-794.44068	-794.10700
COD	-311.67316	114.15737	-311.52276	-311.74483	-311.58602
**INT1-I**	-2141.57364	353.33727	-2141.07561	-2141.88596	-2141.38527
**TS1-I**	-2141.57123	351.08443	-2141.07517	-2141.88491	-2141.38602
**INT2-I**	-2141.57540	353.79839	-2141.07830	-2141.88936	-2141.38979
**L**	-848.26211	266.20312	-847.88357	-848.44596	-848.06179
^{t}BuOK·KCl	-1293.28794	87.09433	-1293.18752	-1293.41819	-1293.31046
Ni(0)L	-1019.12963	266.95146	-1018.75300	-1019.31361	-1018.93178
Ni(0)L_2	-1867.48401	533.58961	-1866.70932	-1867.85192	-1867.07669
**R1**	-347.32125	87.20114	-347.21819	-347.39562	-347.28484
**R2**	-646.04275	87.69412	-645.93962	-646.19812	-646.08743
**R3**	-528.00452	159.78707	-527.79026	-528.13188	-527.91087
**R3’**	-1360.63330	239.49200	-1360.29987	-1360.85683	-1360.51822
**COM1-I**	-2513.60203	623.48043	-2512.69111	-2514.12812	-2513.21801
**COM2-I**	-2012.64218	445.21051	-2012.00491	-2013.05652	-2012.41815
**TS2-I**	-2012.62742	444.98762	-2011.99090	-2013.04014	-2012.40251
**INT3-I**	-2012.66127	445.83758	-2012.02344	-2013.07362	-2012.43468
**TS3-I**	-2012.63585	445.33742	-2011.99799	-2013.04842	-2012.40939
**INT4-I**	-2012.65330	445.71277	-2012.01517	-2013.06682	-2012.42760
**INT5-I**	-2012.64499	445.22969	-2012.01081	-2013.05843	-2012.42352
**INT6-I**	-3373.32309	687.07914	-3372.32567	-3373.96096	-3372.96688
**TS4-I**	-3373.30716	685.42661	-3372.31429	-3373.94488	-3372.95558
**INT7-I**	-3373.33475	686.05636	-3372.34539	-3373.97521	-3372.98993
**TS5-I**	-3373.29711	685.97367	-3372.30708	-3373.93636	-3372.95035
**INT8-I**	-3373.33874	687.22977	-3372.34701	-3373.97715	-3372.98949
**INT9-I**	-2030.05130	489.16496	-2029.34936	-2030.46417	-2029.76190
**P1**	-1010.82924	221.73681	-1010.52978	-1011.05760	-1010.75358
^{t}BuOB(Pin)·KF	-1343.26093	196.86495	-1342.99518	-1343.48619	-1343.21506
**COM2-VI**	-2012.63841	444.82554	-2012.00375	-2013.05280	-2012.41726
**TS2-VI**	-2012.61781	444.74974	-2011.98318	-2013.03066	-2012.39512
**COM2-I'**	-2012.63923	444.62206	-2012.00492	-2013.05325	-2012.41807
**COM2-VI'**	-2012.63516	444.44644	-2012.00206	-2013.04914	-2012.41527
**$COM2_{R2R3-a}$**	-2193.31532	517.60515	-2192.57037	-2193.78401	-2193.03921
**$COM2_{R2R3-b}$**	-2193.31698	517.36311	-2192.57199	-2193.78503	-2193.04013
**$COM2_{R1R3-a}$**	-1894.59172	516.98303	-1893.84592	-1894.97694	-1894.23104
**$COM2_{R1R3-b}$**	-1894.58570	516.62967	-1893.84347	-1894.97047	-1894.22845
**$COM2_{R1R3-c}$**	-1894.59133	516.81439	-1893.84632	-1894.97666	-1894.23155
**$COM2_{R1R3-d}$**	-1894.58669	516.37652	-1893.84428	-1894.97167	-1894.22944

**TS2$_{R2R3-b}$**	-2193.28460	517.17341	-2192.54095	-2193.75185	-2193.00841
**TS2$_{R1R3-c}$**	-1894.56261	517.03755	-1893.81501	-1894.94679	-1894.19880
**COM2$_{R2R3'-a}$**	-3025.94817	597.26053	-3025.08282	-3026.51234	-3025.64844
**COM2$_{R2R3'-b}$**	-3025.94482	596.95568	-3025.08099	-3026.50916	-3025.64695
**TS$_{R2R3'-a}$**	-3025.90568	597.42801	-3025.04069	-3026.46790	-3025.60440
**COM2$_{R1R3'-a}$**	-2727.22276	596.62342	-2726.35858	-2727.70330	-2726.84053
**COM2$_{R1R3'-b}$**	-2727.21880	596.26753	-2726.35816	-2727.69907	-2726.84013
**COM2$_{R1R3'-c}$**	-2727.21983	596.69413	-2726.35386	-2727.70086	-2726.83611
**COM2$_{R1R3'-d}$**	-2727.21681	596.10942	-2726.35407	-2727.69768	-2726.83640
**COM2$_{R2R3''-a}$**	-3025.91916	594.98488	-3025.06314	-3026.48420	-3025.63036
**COM2$_{R1R3''-a}$**	-2727.18143	594.52504	-2726.32796	-2727.66271	-2726.81157
**COM2$_{R1R3''-b}$**	-2727.18192	594.47159	-2726.32817	-2727.66350	-2726.81204
**TS-R3'**	-1360.59388	238.06046	-1360.26383	-1360.81911	-1360.48407
**R3''**	-1360.59945	238.02002	-1360.27292	-1360.82542	-1360.49431
**INT6-II**	-2540.67197	607.09240	-2539.79653	-2541.21401	-2540.34072
**TS4-II**	-2540.65300	606.39912	-2539.77447	-2541.19362	-2540.31672
**INT7-II**	-2540.68482	607.73190	-2539.80513	-2541.22493	-2540.34696
**TS5-II**	-2540.66123	606.16053	-2539.78589	-2541.20046	-2540.32701
**INT8-II**	-2030.03292	488.89587	-2029.33429	-2030.44582	-2029.74720
BPin-F	-510.64340	116.94601	-510.49130	-510.76923	-510.60930
**TS4-III**	-3373.27304	685.99576	-3372.28006	-3373.91213	-3372.92275
**INT8-V**	-2030.04509	488.38009	-2029.34393	-2030.45909	-2029.75757
**TS5-V**	-2030.00613	488.09102	-2029.30548	-2030.41945	-2029.71840
**TS4-VII**	-2012.62156	444.61778	-2011.98495	-2013.03507	-2012.39734
**INT5-VII**	-2012.68635	446.13100	-2012.04731	-2013.09882	-2012.45864
**TS5-VII**	-2012.66689	445.66934	-2012.02809	-2013.07916	-2012.43916
**INT6-VII**	-2012.68348	445.96249	-2012.04378	-2013.09475	-2012.45384
Ni(II)F_2L	-1218.75811	270.64786	-1218.37920	-1218.99669	-1218.61316
**P5**	-793.92303	174.07437	-793.69002	-794.09345	-793.85444
F_2	-199.32696	1.54673	-199.34411	-199.37995	-199.39211
**COM1-VIII**	-1665.25583	355.29660	-1664.75349	-1665.59623	-1665.09118
**COM1-IX**	-2214.87982	623.19884	-2213.96972	-2215.32192	-2214.41258
**COM1-X**	-1366.53258	355.15627	-1366.02926	-1366.78968	-1366.28347
